# Local effects of acupuncture on the median and ulnar nerves in patients with carpal tunnel syndrome: a pilot mechanistic study protocol

**DOI:** 10.1186/s13063-018-3094-5

**Published:** 2019-01-05

**Authors:** Alexandra Dimitrova, Charles Murchison, Barry Oken

**Affiliations:** Department of Neurology, Oregon Health &Science University, 3181 SW Sam Jackson Park Road, Mail Code CR120, Portland, OR 97239 USA

**Keywords:** Acupuncture, Electroacupuncture, Structural acupuncture, Mechanistic study, Study protocol, Quantitative sensory testing, Nerve conduction studies, Electrophysiology

## Abstract

**Background:**

While acupuncture’s mechanism of action is not fully understood, there is consensus that the nervous system plays a key role in processing its effects. This research is based on the structural theory of acupuncture, which aims to correlate the location of acupuncture points to peripheral nerves, spinal segments, and spinal plexuses. This mechanistic study explores the close anatomical association between the Pericardium meridian/median nerve and the Heart meridian/ulnar nerve in an attempt to produce electrophysiologic data measuring acupuncture’s direct, nerve-specific effect on the underlying nerves. Specifically, the purpose of this research is to use nerve conduction studies (NCSs) and quantitative sensory testing (QST) to assess for any local, nerve-specific effect of three acupuncture modalities on two anatomically distinct nerves in the forearm — the median and ulnar nerves — in subjects with carpal tunnel syndrome (CTS). The choice of CTS as an injured nerve model allows for comparisons between the response in an injured nerve (median) to that of a healthy one (ulnar).

**Methods:**

Subjects with mild to moderate CTS will be randomized to three intervention groups: manual acupuncture and low- and high-frequency electroacupuncture. Each subject will receive two treatments, 1 week apart, to points in the forearm, which overlay the median nerve (Pericardium meridian) or the ulnar nerve (Heart meridian). Acupuncture will be administered in random order to minimize learning effects in sensory testing. During Week 1, baseline NCS and QST (vibration and cold detection thresholds) will be obtained in both nerve territories, followed by acupuncture and post-acupuncture NCS and QST measurements in both nerve territories. During Week 2, repeat baseline QST and NCS measurements will be obtained, followed by acupuncture to points overlying the nerve not treated in Week 1, followed by post-acupuncture NCS and QST measurements in both nerve distributions.

**Discussion:**

This works aims to capture and characterize the local effects of acupuncture on an underlying nerve and compare them to those on a neighboring nerve. Quantifying acupuncture’s effects using physiologic parameters and discrete values could standardize treatment regimens and help assess their therapeutic effect.

**Trial registration:**

ClinicalTrials.gov, NCT03036657. Registered on 30 January 2017. Retrospectively registered.

**Electronic supplementary material:**

The online version of this article (10.1186/s13063-018-3094-5) contains supplementary material, which is available to authorized users.

## Background

Acupuncture is an ancient therapeutic modality, typically involving needling techniques, based on traditional Chinese medicine (TCM) theories of acupoints located along energy channels called meridians. More recently acupuncture has emerged as an important integrative medical treatment, in both the hospital and outpatient settings [1, 2]. In 1998 a National Institutes of Health (NIH) Consensus Development Panel concluded that acupuncture is efficacious in adult post-operative and chemotherapy-induced nausea and vomiting, and that acupuncture is helpful in other conditions, including stroke rehabilitation [3]. More recent evidence-based reviews have shown the therapeutic benefits of acupuncture for chronic low back pain [4, 5], migraine and tension headaches [6, 7], chemotherapy-induced nausea and vomiting [8], and other pain conditions [9].

In spite of acupuncture’s increased acceptance, its mechanism of action remains unknown. There is debate as to whether there is point-specific response in acupuncture or whether it induces a more generalized response, perhaps related to endogenous opioids or placebo. Early research suggested that the effects of acupuncture are systemically mediated by the endogenous opioid system [10–12]. Acupuncture was shown to increase cerebrospinal fluid levels of endorphins, enkephalins, and adrenocorticotropic hormone, and its effects were blocked by the endorphin antagonist naloxone [10, 12]. The immune effects of acupuncture seem to be mediated via increased activity of splenic natural killer (NK) cells and levels of interferon gamma [13]. Acupuncture may also affect the gene expression of neuropeptides and their receptors [14, 15]. However, a non-specific, systemic mechanism cannot explain why acupuncture is conventionally applied in close proximity to the locus of pain and why the analgesic effects of acupuncture are often limited to the ipsilateral side [16, 17].

For many decades acupuncture practitioners and anatomists have attempted to correlate the location of acupuncture points to peripheral nerves, spinal segments, and nerve plexuses [18–24]. This neuroanatomical theory of acupuncture suggests that acupuncture’s effect is mediated via afferent input through the peripheral nervous system, eliciting a reflex at the level of the spinal cord via the sympathetic plexuses and via efferents to the visceral organs and skeletal muscle [25, 26]. Support for this theory may come from the TCM concept of the sensation “DeQi,” which is considered vital for achieving therapeutic results. It has been described as aching, soreness, heaviness/pressure, fullness, warmth, coolness, tingling, numbness, and dull pain at the site of the needle [27] or radiating paresthesias [28]. It appears that this sensation is mediated by the peripheral nerves. In clinical practice patients liken the sensation of needle manipulation to “hitting one’s funny bone,” and neuropathy patients liken acupuncture to the feet to an exacerbation of their neuropathy symptoms. Langevin et al. [29] have suggested that the sensation of DeQi is elicited by fascia manipulation; however, muscle fiber contraction surrounding the needle remains a possibility.

The neurophysiologic testing to support these theories is lacking so far, and further study on the local effects of acupuncture on the peripheral nervous system is needed. There is a consensus, however, that the nervous system is vital in processing the effects of acupuncture [30, 31].

## Methods/design

### Hypothesis

Our general hypothesis is based on the neuroanatomical theory of acupuncture, and we hypothesize that needles placed over a nerve cause nerve-specific changes, as assessed by nerve conduction studies (NCSs), and cold and vibration sensory threshold improvements only in the underlying nerve and its respective sensory territory, and not in another nerve in the same limb which is not being needled. One possible explanation of such needle-induced changes in the underlying nerve is the mechanical (in manual acupuncture) and electrical stimulation (in electroacupuncture) delivered by the needle to the nerve and perineural tissues.

### Study objectives

#### Primary objective/specific aim 1

Characterize the effects of acupuncture on quantitative sensory testing (QST) (cold and vibration sensation thresholds) and NCS parameters in the median and ulnar nerves.

*Hypothesis: Acupuncture will cause a decrease in cold and vibration detection thresholds (improve sensation) in the underlying nerve sensory distribution only. Acupuncture will cause NCS changes characteristic of improved nerve function.*

#### Secondary objectives

##### Specific aim 2

Compare the effects of acupuncture on a diseased nerve (median) to those of a healthy nerve (ulnar), using QST (cold and vibration sensation thresholds) and NCS parameters.

*Hypothesis: There will be a greater change in cold and vibration detection thresholds and NCS parameters in the median nerve compared to the ulnar, due to the lower functional baseline of the median nerve.*

##### Specific aim 3

Compare the effects of manual acupuncture to those of low-frequency and high-frequency electroacupuncture.

*Hypothesis: High-frequency electroacupuncture will have a greater effect on NCS and QST followed by low-frequency electroacupuncture and manual acupuncture, in accordance with traditional practices.*

### Overview

**Fig. 1:**
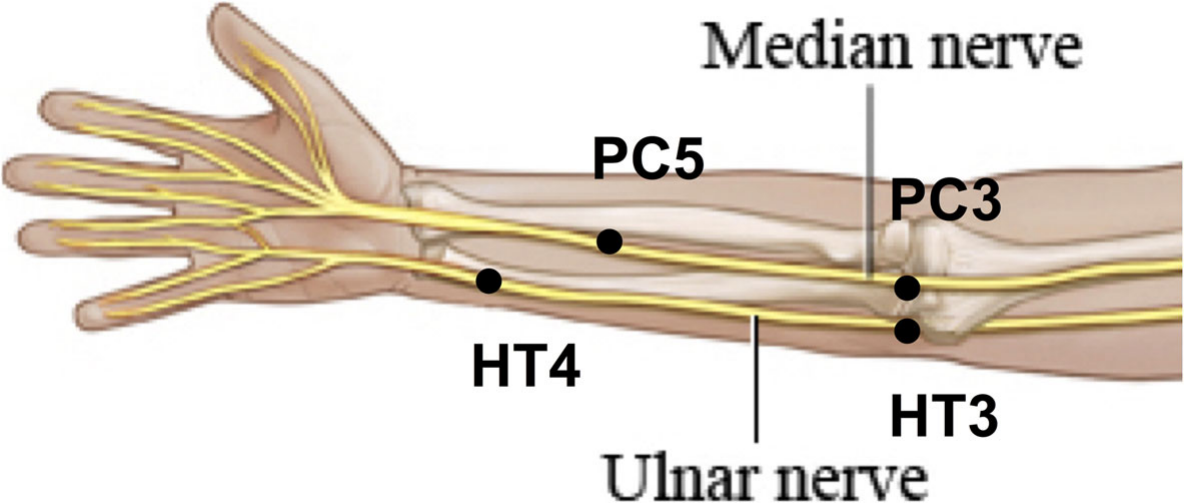
Acupuncture points used

This is a pilot mechanistic study which aims to investigate the local, nerve-specific effects of acupuncture. To accomplish this, acupuncture will be applied to acupoints in the Pericardium and Heart meridians, which are closely associated with the median and the ulnar nerves, respectively (Fig. 1). All subjects will have a diagnosis of carpal tunnel syndrome (CTS).

**Fig. 2:**
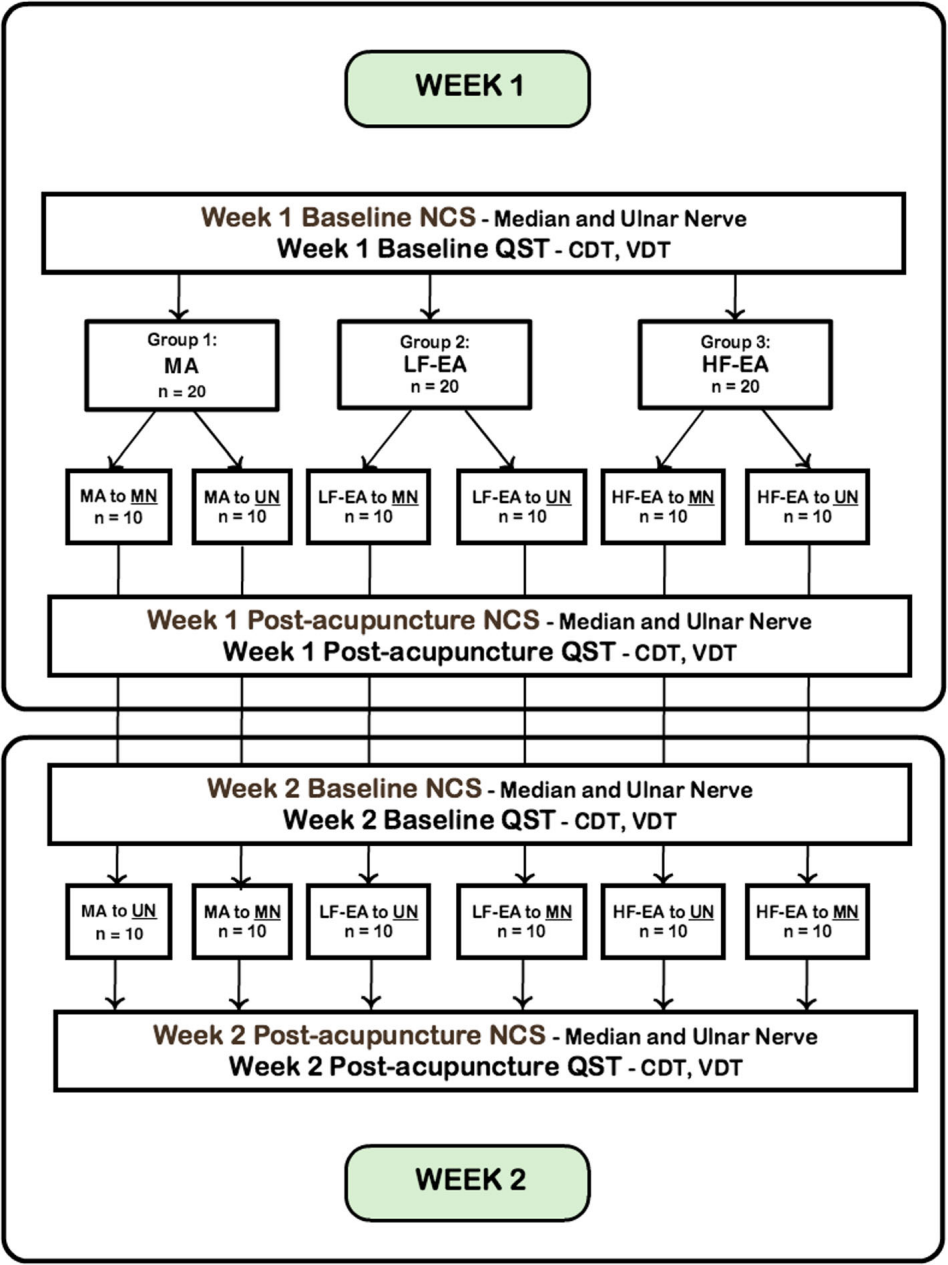
Study procedures flowchart

Subjects will be randomized to one of three intervention groups: manual acupuncture (MA), low-frequency electroacupuncture (LF-EA), and high-frequency electroacupuncture (HF-EA). The intervention will be acupuncture (MA, LF-EA, or HF-EA), and the outcome variables will be post-acupuncture NCSs of the median and ulnar nerves and QST measurements in the palmar median and ulnar nerve territories. Specifically, the QST modalities tested will be cold and vibration detection thresholds (Fig. 2). In each group there will be two treatments in random order, in which acupuncture will be applied to the median nerve/Pericardium meridian and the ulnar nerve/Heart meridian, 1 week apart.

#### Rationale

CTS, which is median neuropathy at the wrist, was chosen as a model of nerve injury, because the location of nerve injury is known and because of its close anatomical relationship to the Pericardium (PC) meridian. Acupuncture has shown to be effective for mild-moderate CTS [32–35] with lasting effects on NCS parameters a year post-treatment [36]. Our recent systematic review of successful randomized controlled trials (RCTs) using acupuncture for the treatment of CTS revealed that all studies employed numerous acupuncture points on the PC meridian, in proximity to the site of injury and in close association with the median nerve [32–35]. The anatomical connection of the acupuncture point PC6 and the median nerve has been well established both in cadavers [37] and in live subjects using ultrasound [38, 39], most strikingly with a documented case of the acupuncture needle piercing through the median nerve sheath, with absence of any symptoms in the subject [39]. These studies highlight the effects of acupuncture in the PC meridian on the underlying median nerve.

The ulnar nerve will serve as a healthy control. It was chosen due to its proximity to the median nerve and close association with the Heart (HT) meridian [34]. The primary objective of this study is to determine whether acupuncture delivered to the PC meridian/median nerve has effects only on the median nerve, or whether these effects are carried over to the neighboring ulnar nerve. Conversely, acupuncture to the HT meridian/ulnar nerve will be tested for local effects on the ulnar nerve and also on the neighboring median nerve. A secondary goal of using the ulnar nerve as a control is to compare the effects of acupuncture on a diseased nerve (median) to those on a healthy nerve (median), using NCS and QST as outcomes. The third goal of this study is to explore the preceding acupuncture/nerve correlations using three different acupuncture modalities—MA, LF-EA, and HF-EA—and to compare their effects on NCS and QST.

#### Study population and enrollment procedures

**Fig. 3:**
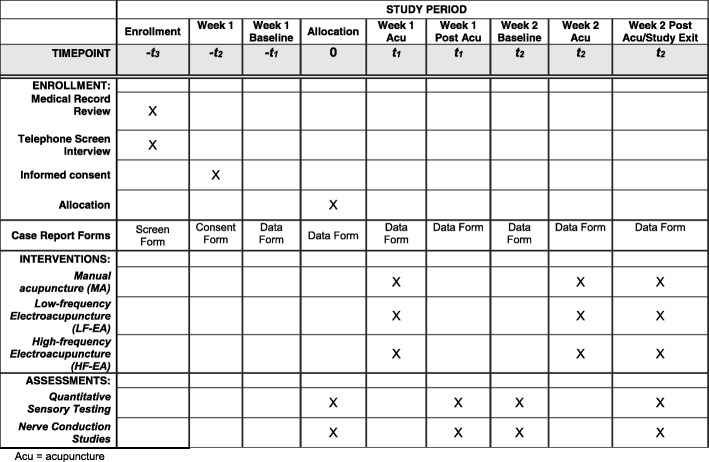
SPIRIT schedule of enrollment

The study’s protocol has been approved by the Oregon Health & Science University (OHSU) Institutional Review Board (IRB). The Standard Protocol Items: Recommendations for Interventional Trials (SPIRIT) checklist is provided as Additional file 1. The projected enrollment is 60 subjects, male and female, ages 18–75. Subjects with mild or moderate CTS, who had an electromyography (EMG) performed at OHSU’s EMG lab within the past 2 years, will be selected after EMG record review. The diagnosis of CTS will be made in accordance with the OHSU EMG lab’s guidelines [40–42]. For details on the specific EMG criteria, see Table 1. Subjects will be approached, using an IRB-approved telephone script, with information about the study and screened for inclusion/exclusion criteria (Table 1). The exclusion criteria largely aim to avoid patients with sensory deficits (besides CTS symptoms) or cognitive impairments, who may be unable to comply with or tolerate QST and NCS. If a potential subject is determined to be eligible, the Principal Investigator (PI) will answer all his/her questions, inform the subject that he/she may withdraw consent at any point, and obtain written informed consent for the study. Following enrollment, the subject will undergo Week 1 baseline QST and NCS testing. If the subject meets any of the secondary exclusion criteria, he/she will exit the study prior to any randomization and treatment (Fig. 3). All other subjects will proceed to randomization.

**Table 1:** Inclusion/exclusion criteria

Inclusion criteria	Age: 18–75
	Sex: M, F
	CTS diagnosis: EMG at OHSU’s EMG lab within the past 2 years, meeting criteria for mild-to-moderate CTS
	CTS symptoms: Present for at least 3 months [42]
	EMG inclusion criteria: mild-moderate median entrapment neuropathy (CTS) defined as meeting any of the following 3 conditions [40, 41]: 1. Prolonged distal median sensory AND/OR motor latency 2. Reduced median sensory nerve action potential (SNAP) amplitude by no more than 50% 3. Amplitude of the compound muscle action potential (CMAP) recorded from abductor pollicis brevis (APB) > 50% of normal
Exclusion criteria	Conditions in which acupuncture/electroacupuncture may be contraindicated: - Coagulopathy/current anti-coagulation treatment - Epilepsy - Cardiac pacemaker - Pregnancy - Presence of any skin condition in the arm, such as dermatitis, bruises, other skin lesions
	Conditions in which QST testing may be contraindicated: - Significant cognitive impairment such as diagnosis of Alzheimer’s disease or mental retardation or any other condition interfering with alertness and/or attention - Hospitalization for anxiety or depression in the past 3 months - Current psychiatric diagnoses (other than anxiety or depression) - Illicit drug use in the past month - Current alcohol abuse (> 2 drinks/day) - History of significant neurological disease which may affect sensation: strokes, multiple sclerosis, or spinal cord disorder - Change in neuropathy medications within the past 2 months - Change in opioids, benzodiazepines, selective serotonin reuptake inhibitors (SSRIs), or other sedating medications in the past 2 months
	Conditions which predispose to generalized neuropathy: - Diabetes mellitus - Uncorrected hypothyroidism - Past chemotherapy treatment
	Other contraindications: - History of wrist or elbow fracture, past arm trauma, loss of fingers, scarring - History of carpal tunnel release surgery or any other surgery on the arm or shoulder - History of arthritis in the hand and elbow - Use of any investigational drugs within the previous 6 months
	EMG exclusion criteria; presence of severe CTS, defined as: 1. Absent SNAP recorded from the second digit AND/OR: 2. The amplitude of the CMAP recorded from the ABP muscle is less than 50% of normal (< 2.5 mV) [40, 41] Other EMG exclusion criteria: • Presence of ulnar neuropathy, peripheral neuropathy, or radiculopathy • Presence of Martin-Gruber anastomosis
Secondary exclusion criteria	Following Week 1 baseline QST and NCS measurements: - Severe CTS symptoms leading to inability to tolerate acupuncture or QST - Inability to tolerate NCS or QST - Failure to comply with QST due to inattentiveness, etc. - Hyperalgesia or hypoalgesia on QST - Presence of Martin-Gruber anastomosis - Presence of severe CTS - Pure motor median neuropathy - Ulnar neuropathy

*CTS* carpal tunnel syndrome, *OHSU* Oregon Health & Science University, *QST* quantitative sensory testing

#### Randomization and blinding procedures

Subjects who pass the secondary exclusion criteria will be randomized to MA, LF-EA, or HF-EA, using a specially designed randomization program. Stratified randomization will be performed based on 2 × 2 categories, where one category is sex (M/F) and the other is projected median age split of 56, based on pilot data. The site of initial intervention (median vs ulnar nerves) will be also assigned as part of the randomization. Roughly 50% of the subjects will undergo needling over median nerve-associated points first, and the other 50% over the ulnar nerve points first, so that learning effects from QST will be evenly distributed (Fig. 2). Randomization will be conducted by the study acupuncturist, and all outcome assessors will be blinded to it.

#### Baseline procedures in detail

##### Baseline quantitative sensory testing (QST) measurements

QST will be performed using the Computer Aided Sensory Evaluator (CASE) IV system (WR Electronics, Minneapolis, MN, USA). At baseline, subjects will be tested for vibration detection threshold (VDT) and cold detection threshold (CDT).

##### Vibration detection threshold

Vibration stimuli will be delivered as 25 discrete levels ranging from 0.0 to 350 μm of displacement, based on previously established “just noticeable difference” (JND) values [43, 44]. Each stimulus is presented with an exponential onset and turns off with an exponential decay, in order to eliminate the touch-pressure artifact, which is caused by an instantaneous on/off. Stimulation will be delivered to the finger pads of the second (median nerve territory) and fifth digits (ulnar nerve territory), with the hand on an even surface and the palm facing up.

##### Cold detection threshold

The thermal stimulator uses a four-degree-per-second ramp up and down and is typically operated in a range from 8 to 50 °C, with an accuracy of 0.25 to 1.25 °C, depending on temperature. For high-magnitude thermal (cooling) stimuli, the absolute temperature is limited to 8 °C. Thermal stimuli are approximately 6 s in duration. CDT will be assessed over the thenar eminence and hypothenar eminence with the hand on an even surface, palm facing up [43, 44].

##### The 4-2-1 stimulus presentation algorithm for VDT and CDT assessment

The 4-2-1 stimulus presentation algorithm was developed by Dyck et al. [45] as a more time-efficient alternative to forced choice, as it enables investigators to assess the sensory threshold in a given sensory modality in 2–5 min in most cases.

Testing will begin at an intermediate level (level 13 of 25). The stimulus will be increased (if not felt) or decreased (if felt) in four steps to the point of turnaround (felt at the higher level when not felt at lower levels, or not felt at the lower level when it had been felt at the higher level). After the first turnaround, stepping will be in steps of two. After the second turnaround, stepping will be in steps of one. A total of 20 stimulus events will be used, with five of them being randomly distributed null stimuli. If three consecutive failures are observed at level 25, testing will be terminated, and the subject will be classified as insensitive. If three consecutive successes are observed at level 1, testing will be terminated, and the subject will be classified as supersensitive. Five null stimuli will be randomly interspersed among 15 non-null stimuli. A positive response (indicating perception) to more than one null stimulus will abort the program. The subject will be reinstructed, and the test will be rerun. Three failures (due to spurious answers to null stimuli) when the test is rerun twice after the test is initially aborted will indicate that the algorithm could not be used for this subject, and the subject will be excluded from further participation in the study.

##### Baseline nerve conduction studies (NCSs)

Baseline nerve conduction studies will be performed in accordance with standard OHSU EMG laboratory clinical practices, based on well-established guidelines, as described by Kimura [46]. The NCSs will be performed using the Dantec Keypoint® G4 Workstation (Natus Medical Incorporated, Pleasanton, CA, USA). The subject’s skin temperature will be maintained above 32 °C, using a heat lamp as needed to warm up the arm. Specifically, the following NCSs will be performed.

##### Median nerve conduction study

The NCS parameters measured will be the sensory nerve action potential (SNAP) amplitude from the second digit, the compound muscle action potential (CMAP) amplitudes from the abductor pollicis brevis, the motor and sensory distal latencies, and the sensory and motor nerve conduction velocities in the forearm (from the elbow to the wrist).

##### Ulnar nerve conduction study

The NCS parameters measured will be the SNAP amplitude from the fifth digit, the CMAP amplitudes from the abductor digiti minimi (ADM), the motor and sensory distal latencies, and the sensory and motor nerve conduction velocities in the forearm (from the elbow to the wrist).

##### Acupuncture intervention

Subjects who meet the NCS criteria for mild-moderate CTS and undergo successful baseline QST testing will be randomized to three acupuncture modalities: MA, LF-EA, or HF-EA (Fig. 2). Acupuncture will be administered by an experienced acupuncturist, using sterile single-use MAC acupuncture needles (0.22 × 25 mm and 0.22 × 40 mm; TianJin Haing Lim Sou Won Medical Equipment Co, Ltd., South Korea). Needling depths will be determined by the acupuncturist in accordance with standard practice. The median nerve-associated acupoints used will be PC3 and PC5. The ulnar nerve-associated acupoints used will be HT3 and HT4 (Fig. 1).

**Fig. 4:**
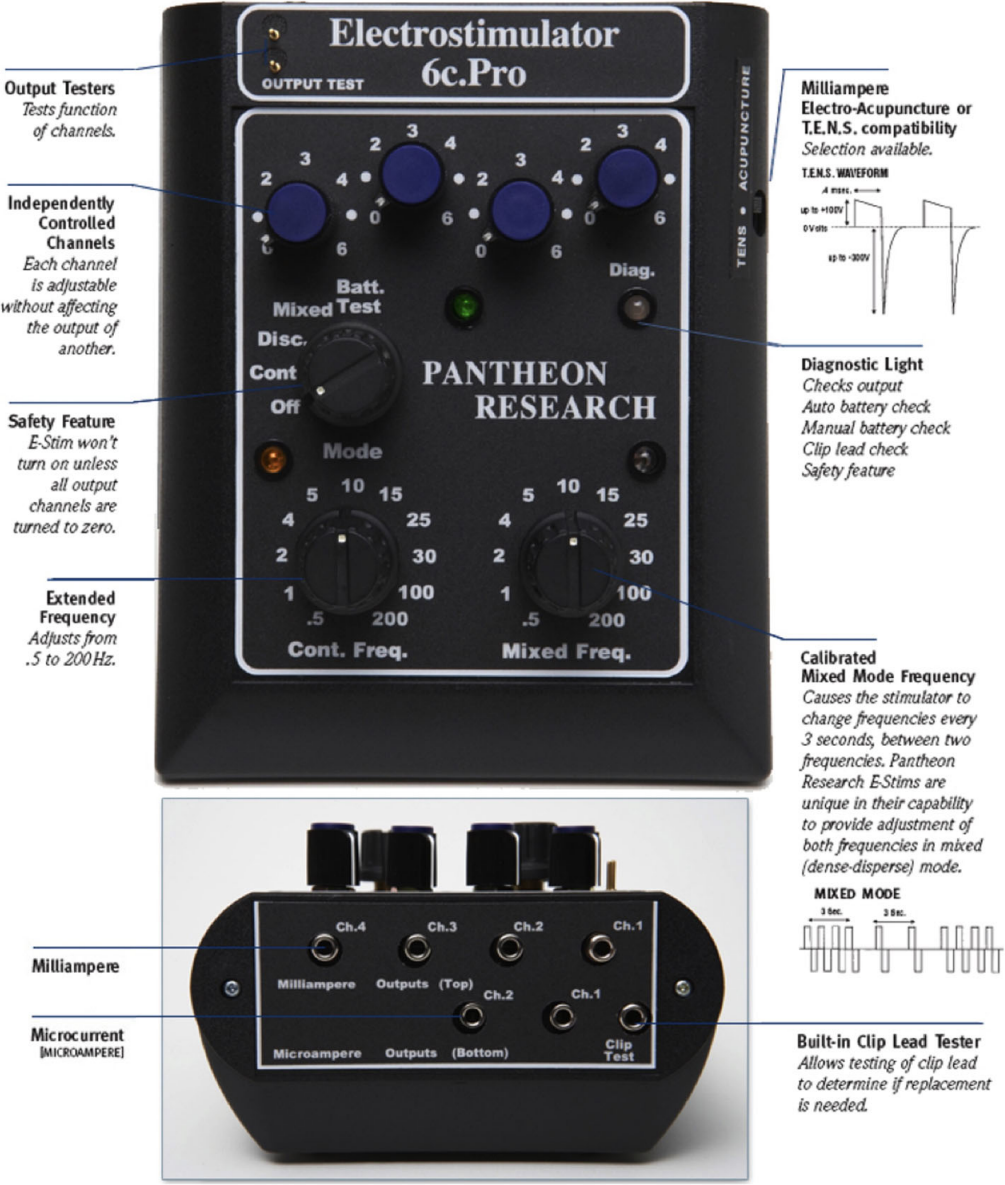
Pantheon 6c.Pro device

In the MA group, manual stimulation will be applied to the needles for 30 s every 5 min. Electroacupuncture will be performed using an electrical device (Electrostimulator 6c.Pro, Pantheon Research, Venice, CA) with insulated cable clamps connected to the acupuncture needles (Fig. 4). In both electroacupuncture modalities the pulse duration will be 60 μs, on a continuous frequency. For *LF-EA*, a frequency of 4 Hz will be used; for *HF-EA*, a frequency of 100 Hz. The stimulus intensity will be increased and readjusted every 5 min, so that the patient feels the stimulation strongly but not painfully (2–8 mA). The acupuncture treatment will last 20 min in each of the three groups.

The two acupuncture interventions will be carried out 1 week apart. There is no general consensus on what constitutes a washout period for acupuncture, as its effects have been difficult to characterize and quantify. A 1-week washout period is widely accepted in crossover studies [47–49], and this period was chosen for this study as well. To minimize any washout effects, a second set of baseline measurements will be conducted at the beginning of Week 2 (Fig. 2).

##### Post-acupuncture measurements

Following 20 min of acupuncture, post-acupuncture CDT and VDT will be obtained using the same protocol as for the baseline measurements. The needles will be left in place. Following post-acupuncture QST, the needles will be removed and post-acupuncture NCS will be performed immediately after, using the same protocol as for the baseline NCS.

#### Outcome measures

The outcome measures will be pre- and post-acupuncture CDT and VDT and pre- and post-acupuncture NCS parameters including median and ulnar nerves’ CMAP amplitude, CMAP area, SNAP amplitude, motor and sensory distal latencies, and motor and sensory nerve conduction velocities.

#### Blinding

Subjects will be blinded as to which nerve is being targeted during each session. It is possible that a subject may possess a sufficient knowledge of anatomy to figure out which nerve is being targeted. All investigators will be blinded to the acupuncture intervention, which will be delivered by a licensed acupuncturist. The investigator collecting QST data will be blinded to the acupuncture intervention. A screen will separate this investigator from the subject and he/she will not have a view of the subject’s arm, needles, or QST probes. The PI will only conduct the NCS and will not be involved in the QST measurements. The PI will enter the room to measure the NCS after the acupuncture treatment and the QST measurements have been completed.

#### Data integrity, safety, and confidentiality

The PI will conduct an IRB-approved medical record review and initiate the confidential study screening form, which will be further completed by the research assistant during a telephone screening interview, using an IRB-approved telephone script. To ensure confidentiality, from the time of screening, only a 5-letter name code will be used to identify the subject. No study forms (besides the consent form) will contain a subject’s name. A key to the 5-letter codes and subject identities will be kept in a password-protected document on OHSU’s database, which requires network and workstation login. All methods to maintain subject confidentiality will adhere to Health Insurance Portability and Accountability Act (HIPAA) standards. HIPAA authorization is part of the study consenting process.

All stored electronic data will be kept in password-protected documents on computers protected by OHSU network passwords. Paper data will be stored in a locked secure cabinet, away from public access. Data storage will be compliant with all HIPAA and OHSU IRB regulations. All research materials will be used for research purposes only.

#### Adverse events and data and safety monitoring plan

An independent study monitor will be appointed in accordance with OHSU IRB regulations. The study monitor will be a neuromuscular expert who has no conflict of interest and no direct involvement in the study. The research staff will report any unanticipated problems and adverse events to the PI and the independent monitor. The PI will be responsible for ensuring that all study procedures follow the approved protocol.

The independent monitor will receive a monthly safety report prepared by the PI, which will include information on any adverse events, unanticipated problems, or protocol deviations. Reportable unanticipated adverse events will include infection at the acupuncture needling site, moderate-severe bruising and bleeding at the acupuncture site, moderate-severe pain and discomfort after acupuncture, new onset of weakness or numbness, permanent worsening of CTS symptoms, and any other adverse events. Additionally, serious adverse events such as death, life-threatening adverse events, inpatient hospitalization, or persistent or significant disability/incapacity will be immediately reported to the study monitor and to the IRB. Unanticipated adverse events as well as an inability to tolerate the experimental procedures will result in immediate exit from the study.     

#### Sample size calculation

A mechanistic study of acupuncture, which examines its local, nerve-specific effects using QST and NCSs as outcome measures has not been attempted before. Therefore, we estimated acupuncture’s effects based on larger studies drawn from the literature. Acupuncture-associated changes in sensation effects were derived from the study of Lang et al. [50], an evaluation of manual and electroacupuncture-induced analgesia in the legs. The authors enrolled 24 healthy subjects, 12 men and 12 women, in six groups in a crossover design between manual, low-frequency, and high-frequency acupuncture and were able to observe significant differences in QST (CDT) between the three acupuncture modalities, *p* = 0.0034.

Effect sizes in median nerve conduction velocity (NCV) were calculated as clinically relevant changes from the mean NCV reported by Kimura et al. [51] and Di Guglielmo et al. [52]. Both studies were NCS characterizations specifically in CTS with 105 and 198 patients respectively and publicized in the American Association of Electrodiagnostic Medicine (AAEM) summary statement on electrodiagnostic parameters in CTS [53].

QST testing results from the acupuncture intervention of Lang et al. [50] were observed to be a significant predictor of CDT, with a 0.2 °C change (− 3.6 ± 0.9 °C to − 3.4 ± 0.4 °C) seen in the low-frequency acupuncture-treated legs. As this study was conducted in healthy subjects, we expect to see a greater effect of acupuncture on patients with median neuropathy at the wrist, especially as the acupuncture will be administered locally, with greater potential to affect the underlying nerve. Sample size estimation assumed the variance of the change in QST outcomes due to local acupuncture delivery over the median and ulnar nerves will be comparable to the variance in QST observed by Lang after acupuncture in healthy nerves (σ^2^CDT = 0.696^2^ °C). Under these assumptions, a minimum QST change of 0.35 °C, considered reasonable as an on-treatment effect size in CDT, would be observed as statistically significant at a significance level of α = 0.05 with 80% power with 20 subjects per arm.

#### Statistical analysis plan

Primary statistical analysis will use linear mixed-effect models to evaluate the effects of treatment group and intervened nerve location on the outcome measures described above. A mixed-model design will be used to create an intention-to-treat framework and allow for any missing data. Principal assessment will be on the change in outcomes resulting from acupuncture intervention, calculated as the difference in outcomes before and after treatment. These outcomes will include CDT and VDT, distal motor/sensory latencies, CMAP and SNAP amplitudes, CMAP area and duration, and nerve conduction velocities. Acupuncture characterization will be carried out in the median and ulnar nerves of the arm, with group being a three-level factor based on the type of acupuncture: MA, LF-EA, and HF-EA.

As a principal independent variable, location of the intervention will be a four-level factor encompassing the possible combinations of treated and evaluated nerves. These levels will be median treated and tested (MM), median treated and ulnar tested (MU), ulnar treated and median tested (UM), and ulnar treated and tested (UU). A time variable (Week 1, Week 2) will also be included to test for any effects due to order of the treated nerves. Additional covariates known to be associated with QST and NCS outcomes such as age and gender will be corrected for as well. Group contrasts within the two primary independent variables will be corrected using Tukey’s honestly significant difference (HSD) test with multiple comparisons accounted for with the Holm-Bonferroni correction. Secondary analysis will include treatment evaluations of outcomes within each location of intervention and additional contrasts between the four time points (pre- and post-treatment for both Week 1 and Week 2). Persistence of the acupuncture effects will specifically be assessed by comparing changes in outcomes after the first treatment but before the second session. Statistical analysis will be performed using SAS 9.1 (SAS Institute, Cary, NC, USA).

## Discussion

### Significance and innovation

Although acupuncture has been shown to have therapeutic effects in multiple medical conditions, its mechanism of action remains unknown. Some studies of acupuncture have emphasized its systemic effects (endogenous opioid, immune system), while others have focused on its local effects and anatomic correlations (fascia, structural theories). To date no unifying theory exists which can accommodate the range of disparate research, and there is a clear need for a systematic exploration into the contribution of the structural mechanisms. The lack of understanding of acupuncture’s mechanism of action is a major obstacle to its wider acceptance. The lack of objective physiological measurements of acupuncture’s effect has posed many difficulties in designing acupuncture research studies involving dose response and efficacy.

Our research will address this knowledge gap by measuring the local effects of acupuncture on an underlying nerve by focusing on nerve conduction study (NCS) parameters and quantitative sensory testing (QST) in the territory of that nerve. Additionally, we will isolate these effects by comparing them to those of a neighboring nerve. Quantifying acupuncture’s effects using physiological parameters and discrete values could standardize treatment regimens and measure therapeutic effect in tangible ways. As acupuncture practices vary widely, both in treatment modalities and point selection, the ability to measure direct effects on the peripheral nerves may help compare treatment regimens and standardize practices by developing efficient protocols.

This line of research is expected to substantiate the proposed association between acupuncture meridians and peripheral nerves in the arm and leg and translate the explanatory constructs of meridians and Qi, drawn from TCM into an acceptable western physiologic paradigm of nerve physiologic changes based on redistribution of charge.

While fascia changes have been implicated in chronic pain conditions [54], likely mediated by a local inflammatory response, the analgesic effects of acupuncture cannot be explained by fascia changes alone, without involvement of the nervous system. Langevin and Sherman [31] recently hypothesized that acupuncture causes changes in mechanoreceptors and nociceptive receptors in connective tissue. Recent histological studies of fascia have shown it to be very rich in Ruffini and Pacinian corpuscles and free nerve endings, and it is therefore believed to be involved in proprioception [30]. The proposed research is innovative because it does not seek to disprove or adopt the fascia mechanistic theory of acupuncture, but rather focuses on the peripheral nervous system’s processing of acupuncture as a final common pathway before further mediation and potentiation occur at the level of the spinal cord and cerebrum.

QST and NCS have both been used in acupuncture research before; however, their use has been to assess therapeutic clinical improvements in the patient [36, 50]. These measurements have never been targeted to a particular nerve distribution and used to study anatomical correlations between acupoints/meridians and an underlying peripheral nerve, with the goal of assessing for acupuncture-induced functional changes in the nerve. We hope that this line of research will lead to a cohesive understanding of acupuncture’s mechanism of action, which incorporates the fascia and the peripheral nervous system effects.

## Pitfalls and limitations

### Subjective nature of QST

This research uses outcome measurements (QST parameters) which in part rely on the subjects’ ability to maintain focus over an extended period of time—about 1 h each for baseline and follow-up. If a subject is not paying attention, he/she may miss a subtle cold or vibration stimulus. To increase accuracy, subjects will be offered a stretch and bathroom break after baseline QST measurement and after acupuncture, as well as water and a snack over the course of a session. Subjects will be tested in a quiet environment and extraneous conversation will not be allowed, in order to minimize distractions. The exclusion criteria will not allow for the enrollment of subjects who have medical conditions which interfere with focus and attentiveness (Table 1).

### Lack of QST normative data

This research uses QST in different locations, for which there are no normative data. QST techniques vary widely in the type of equipment used and in location and modality of sensory stimulation. In spite of this, the AAEM has concluded that QST data are reliable and reproducible using different modalities and testing locations [55]. Through months of testing, we have achieved internal consistency in our QST measurements. Additionally, population normative data are less relevant in this case, as the primary focus is on change from baseline within a subject and on group comparisons.

### Learning effect in QST

There is a learning effect in QST—initially subjects have a harder time feeling subtle cold and vibration stimuli, but they become “sensitized” after a few trials. In order to minimize this learning effect, subjects will be tested in random order; half will undergo median nerve acupuncture and the other half ulnar nerve acupuncture during the first week.

### Lack of non-intervention control

This is a mechanistic study which employs three methods of acupuncture delivery. No sham acupuncture or other non-intervention control condition is used; therefore, it does not control for a possible placebo response. This study focuses on local effects and aims to assess the difference in response to three different acupuncture modalities in two arm nerves. Any placebo-caused improvements in QST or NCS will equally affect all studied nerves, thus diluting its effect.

## Trial status

This trial is currently recruiting participants.

## Additional file

Additional file 1: SPIRIT 2013 checklist: recommended items to address in a clinical trial protocol and related documents. (DOC 121 kb)

## Abbreviations

CDT: Cold detection threshold
CMAP: Compound muscle action potential
CTS: Carpal tunnel syndrome
HF-EA: High-frequency electroacupuncture
HT: Heart
LF-EA: Low-frequency electroacupuncture
MA: Manual acupuncture
NCS: Nerve conduction study
PC: Pericardium
QST: Quantitative sensory testing
SNAP: Sensory nerve action potential
TCM: Traditional Chinese medicine
VDT: Vibration detection threshold
